# Using Survival Analysis to Understand Patterns of Sustainment within a System-Driven Implementation of Multiple Evidence-Based Practices for Children’s Mental Health Services

**DOI:** 10.3389/fpubh.2018.00054

**Published:** 2018-03-01

**Authors:** Lauren Brookman-Frazee, Chanel Zhan, Nicole Stadnick, David Sommerfeld, Scott Roesch, Gregory A. Aarons, Debbie Innes-Gomberg, Lillian Bando, Anna S. Lau

**Affiliations:** ^1^Department of Psychiatry, University of California, La Jolla, San Diego, CA, United States; ^2^Child and Adolescent Services Research Center, San Diego, CA, United States; ^3^Department of Psychology, University of California, Los Angeles, Los Angeles, CA, United States; ^4^Department of Psychology, San Diego State University, San Diego, CA, United States; ^5^Los Angeles County Department of Mental Health, Los Angeles, CA, United States

**Keywords:** evidence-based practices, sustainment, survival analysis, administrative claims data, children’s mental health services

## Abstract

Evidence-based practice (EBP) implementation requires substantial resources in workforce training; yet, failure to achieve long-term sustainment can result in poor return on investment. There is limited research on EBP sustainment in mental health services long after implementation. This study examined therapists’ continued vs. discontinued practice delivery based on administrative claims for reimbursement for six EBPs [Cognitive Behavioral Interventions for Trauma in Schools (CBITS), Child–Parent Psychotherapy, Managing and Adapting Practices (MAP), Seeking Safety (SS), Trauma-Focused Cognitive Behavior Therapy (TF-CBT), and Positive Parenting Program] adopted in a system-driven implementation effort in public mental health services for children. Our goal was to identify agency and therapist factors associated with a sustained EBP delivery. Survival analysis (i.e., Kaplan–Meier survival functions, log-rank tests, and Cox regressions) was used to analyze 19 fiscal quarters (i.e., approximately 57 months) of claims data from the Prevention and Early Intervention Transformation within the Los Angeles County Department of Mental Health. These data comprised 2,322,389 claims made by 6,873 therapists across 88 agencies. Survival time was represented by the time elapsed from therapists’ first to final claims for each practice and for any of the six EBPs. Results indicate that therapists continued to deliver at least one EBP for a mean survival time of 21.73 months (median = 18.70). When compared to a survival curve of the five other EBPs, CBITS, SS, and TP demonstrated a higher risk of delivery discontinuation, whereas MAP and TF-CBT demonstrated a lower risk of delivery discontinuation. A multivariate Cox regression model revealed that agency (centralization and service setting) and therapist (demographics, discipline, and case-mix characteristics) characteristics were significantly associated with risk of delivery discontinuation for any of the six EBPs. This study illustrates a novel application of survival analysis to administrative claims data in system-driven implementation of multiple EBPs. Findings reveal variability in the long-term continuation of therapist-level delivery of EBPs and highlight the importance of both agency and workforce characteristics in the sustained delivery of EBPs. Findings direct the field to potential targets of sustainment interventions (e.g., strategic assignment of therapists to EBP training and strategic selection of EBPs by agencies).

## Introduction

In response to a national call for an increased delivery of evidence-based practices (EBPs) in routine-care settings to improve the quality of care (1–4), mental health systems have increasingly mandated or incentivized the implementation of EBPs. As of 2014, more than 20 states have implemented evidence-based mental health therapies or medication practices either directly or through contracts with other organizations (5–8).

Evidence-based practice implementation requires substantial investments to support the mental health workforce (9, 10). Such costs are incurred through clinicians’ time spent on attending trainings (i.e., lost revenue for the agency) and costs to facilitate the supervision and fidelity monitoring of newly trained staff, including payments to external consultants or trainers (11). For example, staff training and supervision account for 24 and 17.3%, respectively, of the total costs associated with the implementation of Trauma-Focused Cognitive Behavioral Therapy (TF-CBT) at 10 community mental health (CMH) agencies (11). Other major costs identified included non-billable provider time (11.8%) and implementation-related team meetings (11.0%). In mental health services, workforce costs may be especially high given the complexity of multicomponent psychosocial EBPs, many of which have intensive certification requirements (12). As noted by Proctor et al. (13), these costs are dependent on the complexity, strategy, and setting of an intervention. As such, an even greater investment is needed when multiple complex interventions are rolled out in tandem in a given service system (14).

While there is a growing literature on the factors influencing the initial implementation of EBPs, less is known about what transpires in the years following their adoption (15–18). Failure to achieve long-term sustainment of adopted EBPs results in poor return on investment due to the limited public health impact of initiatives (19–21). It is therefore important to examine patterns of EBP sustainment and to identify agency and therapist factors that are associated with sustainment vs. discontinuation of practice delivery over time and inform the tailoring of implementation strategies.

Lack of EBP sustainment and limited success in future EBP implementation efforts are yoked, and potential barriers to practice sustainment abound. In some service settings, new EBPs tend to be cyclically adopted and de-adopted, based on local, state, or federal requirements, practice “trends,” and other contextual influences. Without effective sustainment, these serial EBP adoption and de-adoption cycles have immediate costs in terms of finances, person hours, and later downstream consequences, such as implementation overload and learned helplessness (22), staff cynicism, and resistance to innovation (23). However, two persistent conditions in public mental health service systems are key drivers of sustainment failures. First, stakeholders at the agency, system, and EBP developer levels have reported staff turnover to be the greatest barrier to sustainment (24). Second, inadequate long-term funding of implementation initiatives is a common challenge to EBP sustainment. In a study by Bond et al. (25) of the sustainment of five EBPs implemented across eight states, barriers to sustainment varied by site: 94% of discontinuing sites identified financial reasons as a major barrier, followed by 47% of discontinuing sites citing workforce factors (e.g., the availability of certified practitioners). By contrast, studying sustainment in the context of long-term stable funding of implementation may help to reveal other provider-level characteristics that promote EBP sustainment. Furthermore, when considering system-level outcomes, it is unclear whether EBP sustainment is maintained at the system level even when providers turn over across agencies or organizational units.

Sustainment has been measured in a variety of ways; moreover, sustainment outcomes may depend on how and when sustainment is assessed (17, 26). Possible sustainment outcomes include reach/penetration (i.e., the extent to which a practice is integrated in a service setting as a proportion of population served or population of providers delivering care) or service volume (i.e., the extent to which a practice is delivered over time in a number of agencies, therapists, children, and units of service) (27). Another major index of sustainment is the extent to which trained therapists continue to deliver an EBP to clients in a system once they are trained to do so.

As previously noted, staff turnover at agencies has been identified as a barrier to sustainment, representing penetration-related losses in training investments at the organization level [e.g., Ref. (28, 29)]. However, it is plausible that workforce investments may continue to yield benefits at the system level to the extent that therapists move between organizational units within a larger system that share fiscal resources. For example, Beidas et al. (10) found that 55% of the staff who left a CMH agency within 1 year of follow-up remained in the public sector system, whereas 35% acquired new jobs in the private sector. These findings suggest that even though EBP-training investments may be lost at the agency level when a staff member leaves the agency, some proportion of this loss may be recaptured within a system when the provider is retained at another unit within the system. The extent to which this occurs has not been studied and offers a complementary sustainment outcome that is particularly relevant to system-driven implementation efforts.

Administrative claims data have been identified as a valuable resource for researchers and policymakers alike to understand the sustainment of mental health policy/program initiatives across agencies (15, 30, 31, 32, 33). Claims data provide an opportunity for a novel application of survival analysis to examine sustainment in the context of large, system-driven EBP implementations. Also known as duration analysis, event history analysis, or failure analysis, among other names, survival analysis is an analytic method used in a variety of fields, ranging from economics to sociology to engineering to measure the length of time until a defined event occurs (34). In mental health research, survival analysis has been used to examine therapist turnover [e.g., Ref. (29)] and client attrition/psychotherapy termination at a clinic [e.g., (35)], but has yet to be harnessed to study EBP sustainment in mental health services. Administrative claims data also provide the opportunity to identify potential factors associated with a sustained EBP delivery. It is plausible, for example, that certain therapist characteristics (e.g., bilingual competence) and case-mix characteristics (e.g., alignment of predominant population diagnosis to EBP) may bode well for a sustained EBP delivery.

## Materials and Methods

### Context

The current study is an exploratory one that applies survival analysis to a novel context—measuring therapists’ sustained delivery of multiple EBPs in the context of a system-driven implementation. Administrative data were collected through the Prevention and Early Intervention (PEI) program in the Los Angeles County Department of Mental Health (LACDMH), the largest county mental health department in the USA (27). The purpose of this study was to use EBP-specific claims data to (1) characterize therapists’ continued vs. discontinued delivery of six EBPs [Cognitive Behavioral Interventions for Trauma in Schools (CBITS), Child–Parent Psychotherapy (CPP), Managing and Adapting Practice (MAP), Seeking Safety (SS), TF-CBT, and Positive Parenting Program (Triple P)] and (2) identify factors associated with a sustained EBP delivery. Consistent with implementation models [e.g., Ref. (26)], organizational and therapist characteristics [e.g., Ref. (25, 36, 37)] as well as case-mix characteristics [e.g., Ref. (15)] have been associated with implementation outcomes.

Beginning in fiscal year 2010–2011 and within the context of a state budget shortfall, LACDMH-contracted agencies and directly operated programs were offered the opportunity for reimbursement for the delivery of a number of evidence-based and community-defined EBPs through the PEI transformation in children’s mental health services. LACDMH furnished initial implementation support (i.e., initial training and consultation) for six EBPs for children and adolescents, including CBITS, CPP, MAP, SS, TF-CBT, and Triple P, which were selected for supported implementation based on both the presenting problems (not diagnosis) targeted and the capacity of the EBP developers to train very large numbers of therapists within a short time frame (38). Table 1 provides a brief summary of these EBPs. Training was ongoing throughout the study time frame; therapists could be trained and begin claiming under PEI at any time within the 19 fiscal quarters. Funding support for PEI training and delivery was also ongoing, as dictated by the California Mental Health Services Act, which was passed by voters in 2004. This is a permanent state-funding stream that can only be terminated or altered by a majority of state voters through a new ballot initiative. However, county plans for fund administration may be subject to change. Generally, therapists received training in some but not all of the six practices. As indicated in a recent paper (39) examining survey responses from a sample of 720 therapists in this county, therapists were trained in an average of 2.42 (SD = 1.04) out of these six possible practices.

**Table 1:** Indicated age range, target problems, and consultation and training requirements for the six EBPs as noted in the PEI Implementation Handbook, Revised July 2016.

	Indicated age range (years)	Target problems	Ongoing consultation	Minimum training required before claiming is allowed	Train-the-trainer allowed?
Cognitive Behavioral Intervention for Trauma in Schools	10–15	PTSD, traumatic stress	Weekly consultation calls for at least 10 weeks are required	2-day on-site	Yes

Child–Parent Psychotherapy	0–6	Trauma, poor attachment	Bi-weekly group consultation calls for 18 months; 6- and 12-month booster trainings are required	Initial 2½ days	No

Managing and Adapting Practice	0–23	Anxiety, conduct, depression, traumatic stress	Twice-monthly consultation calls for 6 months are required (unless trained by an agency-based supervisor)	8 h	Yes

Seeking Safety	13+	PTSD, substance use	Consultation calls are not required	Initial 6 h	Yes

Trauma-Focused Cognitive Behavioral Therapy	3–18	PTSD, traumatic stress	12 consultation calls and a booster training are required	Webinar and initial 2-day in-person	No

Positive Parenting Program	0–18	Disruptive behavior, family dysfunction	Consultation calls are not required	Initial training (1–3 days)	No

### Procedures

The current study extracted administrative PEI claims data for the six initial EBPs supported by LACDMH for implementation in children’s mental health services, spanning 19 fiscal quarters, or approximately 57 months, between fiscal years 2009–2010 (Quarter 4) and fiscal years 2014–2015 (Quarter 2). These data capture the initial rollout through early sustainment period of this EBP implementation effort. Claims for this study were restricted to “psychotherapy” units of service that were delivered to clients under 21 years old (defined as youth by LACDMH), that occurred between May 11, 2010, and December 31, 2014, and that were delivered by therapists who billed at least three psychotherapy claims during this time frame. These data represent 6,873 unique therapists who were employed within 88 unique agencies and billed a total of 2,322,389 psychotherapy claims. Claims were aggregated to the therapist level for the delivery of each practice and for the delivery of any of the six EBPs.

As part of the larger 4KEEPS Project (27), this study was approved by the Institutional Review Board at the University of California, Los Angeles, CA, USA.

### Participants

A total of 6,873 therapists were represented in the extracted claims across the study period. Therapist demographic, professional, and case-mix characteristics derived from the claims data are provided in Table 2.

**Table 2:** Therapist-level demographic, service, case-mix, and agency characteristics.

	Categorical variables		Continuous variables	
	*n*	%	Mean	SD
**Demographics**				
Made first claim in 2010 (early entry control)	2,037	29.6		
**Discipline/type**				
Marriage and family therapist	1,954	28.4		
Rehabilitation professional	1,407	20.5		
Counselor	1,355	19.7		
Social worker	795	11.6		
Trainee	530	7.7		
Other	648	4.4		
Psychiatrist	184	2.7		
Primary language				
English	3,868	56.3		
Spanish	2,392	34.8		
Other	613	8.9		
Ethnicity				
Hispanic/Latino	2,392	34.8		
Other non-Hispanic minority	2,422	35.3		
Non-Hispanic White	2,059	30.0		
**Service characteristics**				
Average number of EBP claims made per active day			1.81	0.83
Average number of clients served per month with EBP			1.38	2.54
Number of agencies at which therapists billed			1.13	0.40
Number of EBPs for which therapist billed			2.18	1.11
**Case-mix characteristics**				
Client race/ethnicity (% of a therapist’s caseload)				
Hispanic			64.13%	29.31%
Other non-Hispanic minority			22.65%	25.01%
Non-Hispanic White			9.40%	15.51%
Client primary presenting problem/admission diagnosis (% of a therapist’s caseload)				
Internalizing disorders: mood or anxiety disorders			42.56%	26.75%
Externalizing disorders: disruptive behavior disorders or ADHD			29.94%	24.26%
Adjustment or other disorders			17.56%	22.40%
Trauma disorders			9.90%	16.06%
Client average age			11.79	3.40
Client gender (% males)			53.66	27.13
Service setting (% of a therapist’s total claims)				
Office (outpatient)			57.28%	37.32%
Home			19.78%	27.02%
School			13.12%	21.36%
Other			9.07%	20.97%
**Agency characteristic**				
Final claim agency having multiple sites	5,158	75.0		

### Measures

All therapist characteristics were derived from the claims data. For each categorical variable, the largest category was selected as the reference group. The following *therapist demographics* were included as categorical predictors in each model: primary language (English, Spanish, other), discipline/type at the time of therapist’s first PEI claim [marriage and family therapist (MFT), rehabilitation professional, counselor, social worker, trainee, psychiatrist, other], and race/ethnicity (Hispanic/Latino, non-Hispanic White, other non-Hispanic minority).

Therapist *service characteristics* included the following continuous predictors: the average number of claims that the therapist billed to PEI per active day, the average number of unique clients served per month, the total number of agencies at which the therapist claimed for one of the six PEI EBPs during the time frame, and the total number of EBPs out of six for which a therapist claimed. An *active day* was defined as a day in which a therapist made at least one claim. The practice for which each therapist made the most claims was included as a predictor in the model examining continued delivery of any of the six EBPs.

Therapist *case-mix characteristics* included the following continuous predictors, which were determined based on the percentage composition of a therapist’s total caseload or total claims during the time frame: client admission diagnosis (percentage of a therapist’s caseload whose admission diagnosis was an adjustment disorder or a disorder other than mood/anxiety, disruptive behaviors, ADHD, or trauma), client ethnicity (percentage of a therapist’s caseload that is Hispanic), and service setting [percentage of a therapist’s claims that take place in an office rather than in field settings (home, school, other community locations)]. In addition, the average client age and client gender (percentage of a therapist’s caseload that is male) were included in the model as continuous variables. We examined the percentage of a therapist’s caseload whose primary diagnosis was an adjustment or other disorder, because these disorders are not explicitly matched to presenting problems targeted by the EBPs; thus, high proportions may relate to discontinuation. Second, we examined the percentage of a therapist’s caseload that is Hispanic, reasoning that therapists who serve a high proportion of the most well-represented ethnic group in the LACDMH child population may be more likely to be retained in the system. Third, we included the percentage of a therapist’s total claims that occurred in the office, because it is reasonable to ask whether providing more office-based or field-based services may relate to sustained EBP delivery.

To assess *agency factors*, agency centralization (multiple sites vs. single site) was included as a predictor in the model. Agency centralization data were obtained from DMH technical site visits in fiscal years 2011–2012 and 2012–2013 (38). Whether or not an agency has multiple sites can also be construed as a binary indicator of agency size.

### Analysis Plan

#### Characterizing Duration of Therapists’ Continued EBP Delivery

The mean and median lengths of delivery (i.e., survival times) were calculated for the delivery of each practice and for the delivery of any of the six EBPs. Kaplan–Meier (KM) survival functions were generated as well, and differences across the six EBPs were determined using the log-rank, Wilcoxon, and Tarone–Ware tests of survival function equality. Since the results of the three tests did not differ, only results from the log-rank test are reported below.

#### Factors Associated with Risk of Discontinuation of Any EBP

A multivariate Cox regression (semi-parametric survival analyses) model was performed to determine the unique contribution of each predictor variable to the sustainment of therapists’ overall EBP practice delivery. The Cox regression model was selected because, as a semi-parametric model, no assumption had to be made about the distribution of the survival time (40). Survival time represented the time elapsed, in units of months, from the time of the therapist’s first claim to the time of the therapist’s final claim for any of the six EBPs. The binary outcome variable was (1) sustained delivery (i.e., censored) vs. (2) discontinued delivery (i.e., failure event). Sustained delivery was right-censored and defined as a continued claiming through the end of available claiming data, which was the fourth fiscal quarter (Q4), or the final 3 months, of 2014, between October 1 and December 31, 2014. Discontinued delivery was defined as *not* claiming for any of the six EBPs during this final quarter of our data. Therapists who “paused” claiming for EBPs over a 3-month period prior to 2014 (e.g., in 2012, 2013, or 2014) but who resumed claims were not considered to experience a discontinuation event.

A single model examining the predictors of the delivery of any of the six EBPs was conducted. The model controlled for whether the therapist began billing for PEI services during the initial rollout period in 2010 (i.e., early entry) or 2011 or later (i.e., later entry). In addition, consistent with the “shared frailty” approach used by Aarons et al. (29), we included agency ID for the last agency at which a therapist claimed as a term in the model, in order to account for the unobserved agency-level random effect, or shared frailty, of therapists nested within an agency (40). Therapists working at the same agency are presumably subject to the same external environment (e.g., agency climate), which suggests that therapists of a single agency would have a “shared” or a “common” value for their frailty, which represents the therapist’s inherent but unmeasured likelihood of experiencing the event of interest (i.e., discontinued delivery) (40). For 88.9% of the therapists, the final agency was the only agency at which the therapist claimed.

Following a test of the proportional hazards assumption of Cox regressions, a few variables violated the assumption of proportionality, which were consequently entered into the model as having time-varying coefficients: the average number of claims made per active day, the number of agencies at which therapists billed to PEI for one of the six EBPs, and the number of EBPs for which therapists billed to PEI. For all categorical variables, the category represented by the largest number of therapists was selected as the reference category.

All analyses were performed using Stata/SE 13.0 (41).

## Results

On average, therapists made 337.9 (SD = 467.09) claims to the six EBPs of interest and delivered these interventions to 22.25 (SD = 28) clients across the 57 months under study. In this sample, 6,111 (88.9%) therapists made psychotherapy claims for at least one of the six EBPs at only one agency, 652 (9.5%) billed at two agencies, 89 (1.3%) at three agencies, 19 (0.3%) at four agencies, and 2 (0.03%) billed at five agencies. Therapists claimed for an average of 2.18 (SD = 1.11) EBPs (range = 1–6) during the time frame of our data. In addition, 2,387 (34.7%) therapists claimed for one practice; 29.7% claimed for two EBPs, 21.1% claimed for three EBPs, 12.1% claimed for four EBPs, and 2.4% claimed for five or six EBPs. Two thousand and thirty-seven therapists (29.6%) made their first PEI claim for these six EBPs in 2010, whereas 1,651 (24.0%), 1,411 (20.5%), 1,068 (15.5%), and 706 (10.3%) therapists began claiming in 2011, 2012, 2013, and 2014, respectively. One hundred and thirty-nine therapists (2.0%) made their final PEI claim for these six EBPs in 2010, whereas 546 (7.9%), 972 (14.1%), 1,364 (19.8%), and 3,852 (56.0%) therapists’ final claim in this dataset occurred in 2011, 2012, 2013, and 2014, respectively. On average, the length of time from a therapist’s first to final PEI claim within the study time frame and parameters was 21.71 months (SD = 16.32). The average age of clients served was 11.79 (SD = 3.40) years. With respect to the final agency at which each therapist delivered services, 5,158 (75.1%) therapists’ final agencies had multiple sites (vs. a single site), and those agencies served an average of 1,899 (SD = 1,633.4) child/youth clients during the time frame of our data. Based on making their first PEI claim on or before December 31, 2010, 2,037 (29.6%) therapists were in the initial cohort of therapists involved at the outset of this system-driven implementation effort.

### Characterizing Continued EBP Delivery

**Figure 1:**
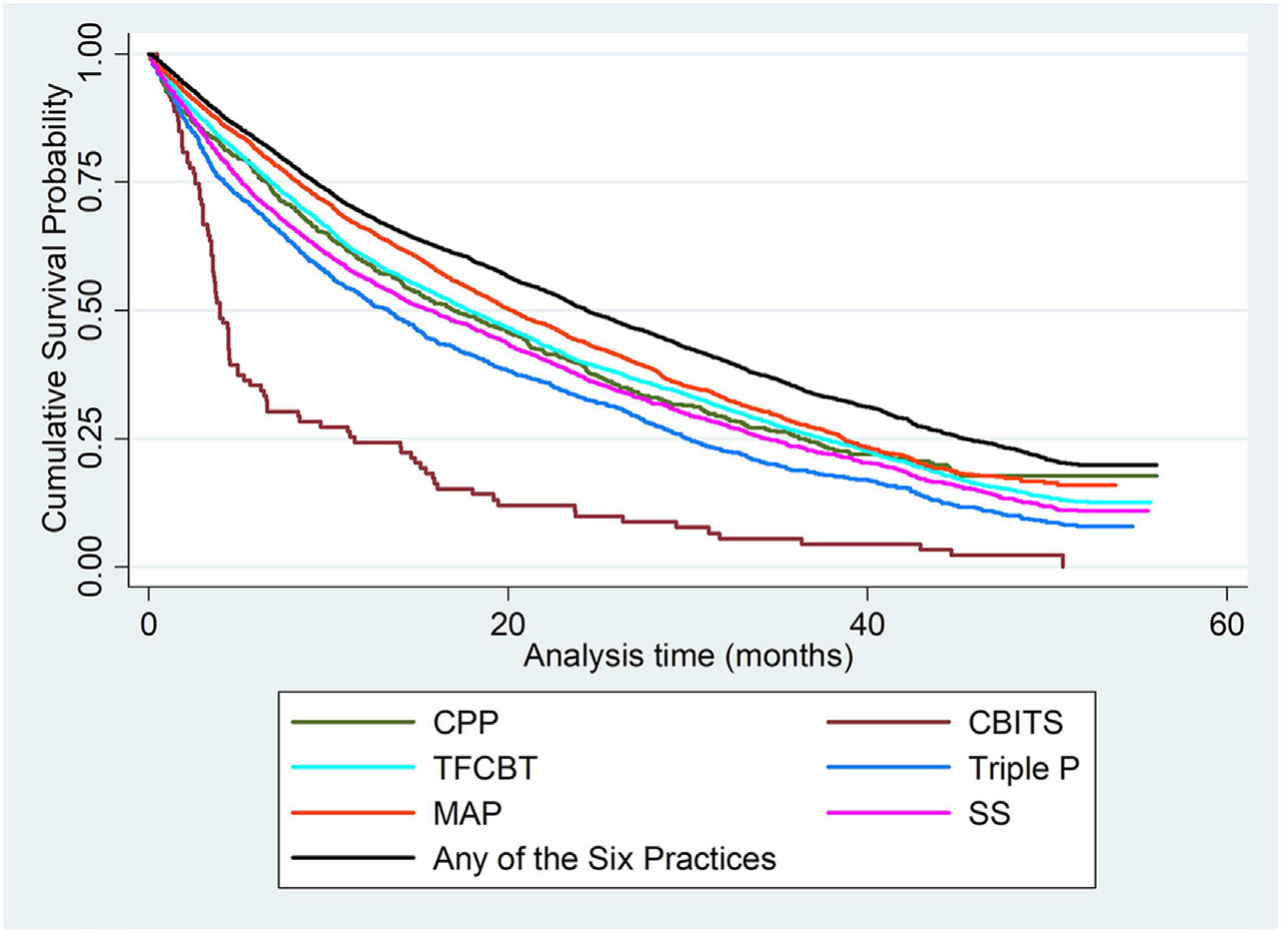
Cumulative Kaplan–Meier survival functions for therapist delivery of each EBP and of any of the six EBPs.

Among all therapists, 2,443 (35.5%) continued delivery of *any* of the six EBPs at the end of the study time frame. Table 3 displays the mean and median survival times, as well as the frequency of discontinued delivery for each practice. Figure 1 shows graphical illustrations of the KM survival functions for therapist delivery of each practice and of any of the six EBPs of interest.

**Table 3:** The mean and median survival times (months) for EBP delivery in the descending order of median survival time.

	Total therapists	Events^a^ (*n*, %)	Censored^b^ (*n*, %)	Mean survival time (months)	Median survival time (months)	Min (months)	Max (months)
Any of the six practices	6,873	4,430 (64.5%)	2,443 (35.5%)	21.73	18.70	0.033	56.10
Managing and Adapting Practices	4,328	2,830 (65.4%)	1,498 (34.6%)	18.16	15.30	0.033	53.80
Trauma Focused-Cognitive Behavior Therapy	4,392	3,239 (73.7%)	1,153 (26.3%)	18.78	14.60	0.033	55.77
Child–Parent Psychotherapy	950	666 (70.1%)	284 (29.9%)	16.53	12.60	0.033	56.10
Positive Parenting Program	1,807	1,479 (81.8%)	328 (18.2%)	16.18	11.43	0.033	54.77
Seeking Safety	3,353	2,447 (73.0%)	906 (27.0%)	16.58	11.20	0.033	55.63
Cognitive Behavioral Interventions for Trauma in Schools	145	142 (97.9%)	3 (2.1%)	8.90	3.97	0.467	50.87

*^a^The number of events represents the number of therapists who discontinued delivery (i.e., made no claims during the final fiscal quarter of 2014)*.

*^b^The percentage of total therapists who continued to deliver during the final fiscal quarter of 2014*.

A visual inspection of Figure 1 indicates that CBITS had a higher risk of discontinuation than the delivery of the other EBPs. The log-rank test of survival function equality revealed significant differences across the six EBPs, *X*^2^ = 207.1, *df* = 5, *p* < 0.001. To identify the specific EBPs that were different from the rest, follow-up log-rank tests were performed to compare the survival curve of each practice to the combined survival curve of the five other EBPs (29). Results revealed that the survival curve for CPP delivery did not significantly differ from the survival curve of the other EBPs (*X*^2^ = 0.02, *df* = 1, *p* = 0.878). CBITS (*X*^2^ = 97.84, *df* = 1, *p* < 0.001), SS (*X*^2^ = 14.81, *df* = 1, *p* < 0.001), and Triple P (*X*^2^ = 58.89, *df* = 1, *p* < 0.001) had a significantly higher risk of delivery discontinuation than the other EBPs, whereas MAP (*X*^2^ = 60.86, *df* = 1, *p* < 0.001) and TF-CBT (*X*^2^ = 4.05, *df* = 1, *p* < 0.05) had a significantly lower risk than the other EBPs. These results align with the patterns visible in Figure 1.

### Factors Associated with Risk of Discontinuation of Any EBP

**Figure 2:**
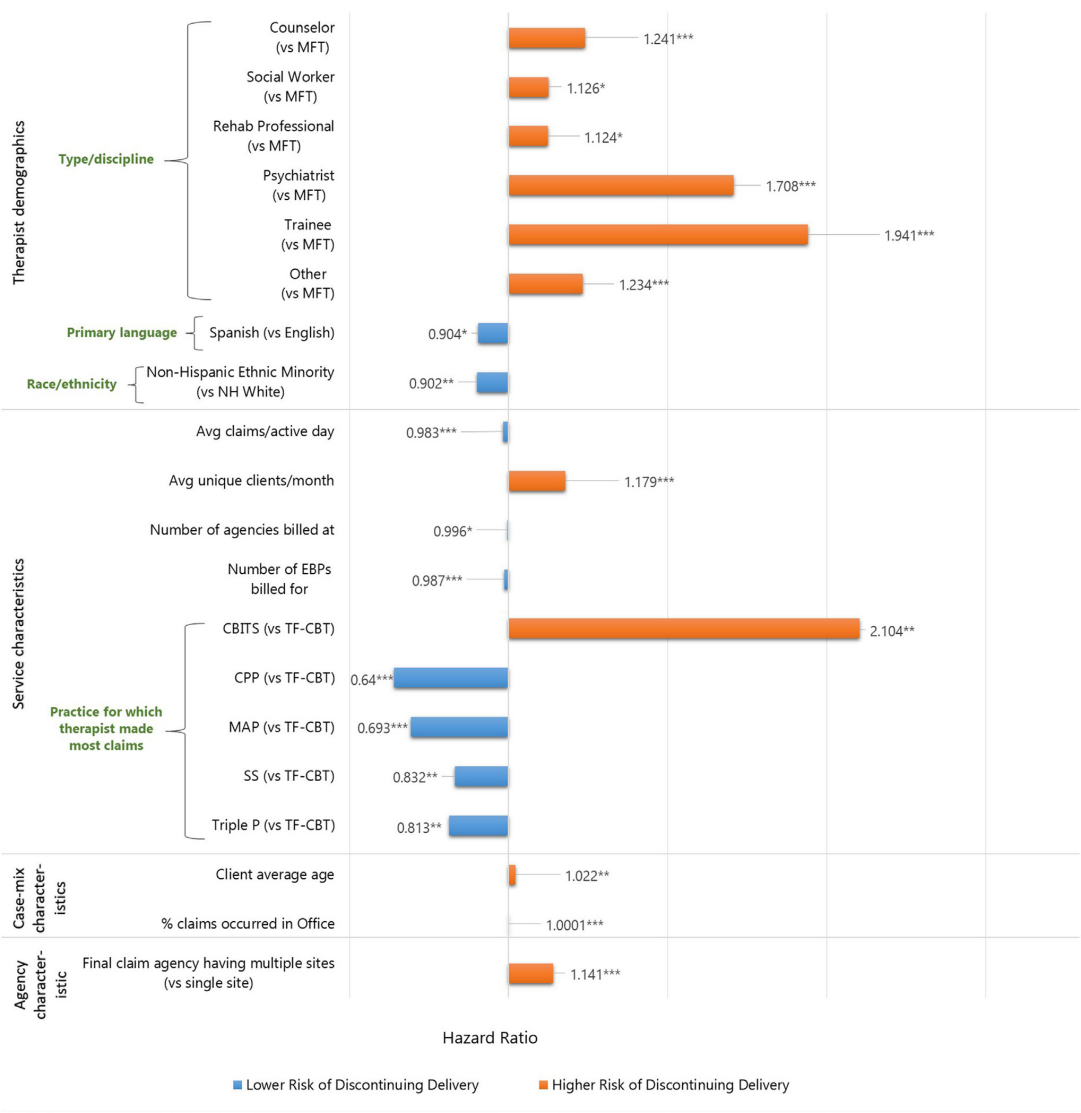
Visual representation of significant predictors in the Cox regression model of discontinued delivery of any of the six EBPs. *p < 0.05, **p < 0.01, ***p < 0.001.

Table 4 and Figure 2 display the results of the multivariate model including predictors of discontinued delivery of any of the six EBPs. For further ease of interpretation, please refer to Figure 2 for illustration of the relative risk of significant predictors. After controlling for whether therapists made their first claim in 2010 or later, results revealed a number of variables to be significantly associated with a risk of discontinued practice delivery. Note that, for categorical variables, hazard ratios indicate how high the risk of discontinuing delivery is for a therapist in one group compared to a therapist in another group, if all other variables were held constant. For continuous variables, hazard ratio indicates a change in the risk of discontinuing delivery if the variable/predictor of interest is increased by one unit (42). For example, the hazard ratio of 0.984 for the average number of daily claims indicates that, for each additional claim made per active day and holding other variables constant, the risk of discontinuing delivery is 0.984 times *lower* (or 1.6% lower) than the risk of discontinuing delivery for a therapist who makes one fewer claim per active day. In the same example, for a therapist who makes 10 more claims per active day—and holding all other variables constant—the relative risk is (0.984)^10^ = 0.851, or a 14.9% *lower* risk of discontinuing delivery.

**Table 4:** Cox regression model for therapists’ discounted delivery of any of the six Prevention and Early Intervention (PEI) EBPs.

	HR	SE
Later entry control (reference = early entry)	1.681***	0.060
**Therapist demographics**		
Therapist type/discipline (reference = marriage and family therapist)		
Counselor	1.241***	0.057
Social worker	1.126*	0.059
Rehabilitation professional	1.124*	0.052
Psychiatrist	1.708***	0.149
Trainee	1.941***	0.129
Other (e.g., Case Manager, Psychologist, etc.)	1.234***	0.072
Therapist primary language (reference = English)		
Spanish	0.904*	0.041
Other	1.032	0.055
Therapist ethnicity (reference = Non-Hispanic White)		
Hispanic	0.950	0.047
Other non-Hispanic minority	0.902**	0.034
**Therapist service characteristics**		
Average number of claims made per active day	0.983***	0.001
Average number of clients served per month	1.179***	0.007
Number of agencies at which therapists billed to PEI	0.996*	0.002
Number of evidence-based practices for which therapists billed to PEI	0.987***	<0.001
Practice for which therapist made the most claims (reference = Trauma-Focused Cognitive Behavior Therapy)		
Cognitive Behavioral Interventions for Trauma in Schools	2.104**	0.51
Child–Parent Psychotherapy	0.640***	0.059
Managing and Adapting Practice	0.693***	0.027
Seeking Safety	0.832**	0.045
Positive Parenting Program	0.813**	0.057
**Case-mix characteristics**		
Client ethnicity (% of caseload that is Hispanic)	0.999	<0.001
Client admission diagnosis (% of caseload that is adjustment or other disorder)	0.999	<0.001
Client average age	1.022**	0.007
Client gender (% of caseload that is male)	1.001	<0.001
Service setting (% of claims that occurred in the office)	1.0001***	<0.001
**Agency characteristic**		
Final agency having multiple sites (reference = single site)	1.141***	0.042

*HR, hazard ratio*.

*p < 0.05, **p < 0.01, ***p < 0.001.

### Therapist Demographic Characteristics

Counselors, social workers, rehabilitation professionals, psychiatrists, trainees, and therapists of other disciplines were at 24.1, 12.6, 12.4, 70.8, 94.1, and 23.4% *higher* risk, respectively, of discontinuing practice delivery than were MFTs. Therapists with Spanish as their primary language exhibited a 9.6% *lower* risk of discontinuing delivery than therapists whose primary language was English. In addition, therapists who identified as other non-Hispanic Minority demonstrated a 9.8% *lower* risk of discontinuing practice delivery, compared to non-Hispanic White therapists.

### Therapist Service Characteristics

Each additional *claim made per active day* was associated with a 1.7% *decreased* risk of discontinuing delivery of any EBP. Each additional *unique client served per month* was associated with a 17.9% *increased* risk of discontinuing practice delivery. Each additional *agency at which a therapist claimed* was associated with a 0.4% *decreased* risk of discontinuing practice delivery. Each additional *EBP claimed for* in total across the study time frame was associated with a 1.3% *decreased* risk of discontinuing practice delivery. Therapists who most frequently billed for CPP, MAP, SS, and TP exhibited 36, 30.7, 16.8, and 18.7% *lower* risk, respectively, of discontinuing delivery of any of the EBPs, compared to therapists who primarily billed for TF-CBT. Therapists who primarily billed for CBITS exhibited a 110.4% higher risk of discontinuing any EBP delivery, compared to those who primarily billed for TF-CBT.

### Case-Mix Characteristics

With respect to case-mix composition, neither the percentage of a therapist’s caseload that is Hispanic nor the percentage of a therapist’s caseload that presented with an adjustment or other disorder was significantly associated with an increased or a decreased risk. The average age of a therapist’s clients was significantly associated with the therapist’s risk of discontinuing practice delivery: each additional year of clients’ average age was associated with a 2.2% *increased* risk. The proportion of male clients on a therapists’ PEI caseload was not significantly associated with a risk of discontinuing practice delivery. With respect to service setting, the percentage of a therapist’s total claims that were made in the office was associated with a 0.01% *increased* risk of discontinuing practice delivery.

### Agency Characteristic

A therapist whose final claim was made at a *multisite agency* exhibited a 14.1% *higher* risk of discontinuing delivery than a therapist whose final claim was made at a single-site agency.

## Discussion

This study highlights a novel application of survival analysis to understand EBP sustainment using administrative claims data to track system-level sustainment of six EBPs over 19 fiscal quarters. Administrative claims were made by therapists delivering EBPs in the context of a system-driven, fiscally mandated implementation of EBPs (i.e., the PEI transformation); PEI funding was available throughout the study time frame. Results revealed that the average survival time for any of the six EBPs within the 57-month study time frame was 21 months, with the average survival time for individual EBPs differing significantly with a range from 9 (CBITS) to 19 months (TF-CBT). Overall, therapist demographic, case-mix, and service characteristics, as well as agency characteristics (centralization), were significantly associated with a risk of therapists’ discontinuation of any EBP. Consequently, these conditions may have implications for return on investment in EBP training.

The first aim of this study was to characterize sustained delivery of any of the six EBPs and examine differences by EBP. As shown in Table 3, the mean and median survival times for the delivery of each EBP or of any of the six EBPs were under 2 years, suggesting that our 5 years of claims data allow for interpretable conclusions. As would be expected, the median survival time is lower than the mean, in part because of the presence of positive outlier therapists who have continued to bill for long periods. However, the mean survival time can be tricky to interpret when there are unequal observation times for each therapist; for example, a therapist could have begun delivering an EBP with only a few months of observation time remaining, with a substantial portion of censored data. The mean is therefore dependent on the time frame of this specific study, and the mean time of *actual* usage in the field is likely to be even longer than what is reported. The median survival time is less susceptible to the influence of study time frame and is more reflective of the median time of actual usage.

Relative to a combined survival curve of the delivery of five other EBPs, therapists who primarily delivered CBITS, SS, and Triple P had a higher risk of discontinuation, whereas therapists who primarily claimed for MAP and TF-CBT had a lower risk of discontinuation within the system. It is unsurprising that CBITS exhibited such a high risk for discontinued delivery; it was never adopted widely by therapists, in part due to its limitation as a school-only EBP (38). Indeed, studies have found program leaders to have positive perceptions of MAP due to the wide range of cases or clients that MAP can be used with (39, 43). In addition, MAP, TF-CBT, and CPP require ongoing consultation, which may have implications for their sustained delivery (44). By contrast, SS and TP do not require ongoing consultation.

The current findings are somewhat consistent with findings on volume-based penetration using the same dataset, in which Triple P, CPP, and CBITS had a lower volume of claims, relative to MAP, TF-CBT, and SS (15). However, the present study found that SS had a significantly *higher* risk of delivery discontinuation than the other EBPs, and that CPP risk did not differ significantly from the other EBPs. These differences reflect how the analysis of different types of sustainment outcomes (claims volume/penetration vs. therapist discontinuation) may generate both convergent and divergent findings.

The second aim of this study was to identify factors associated with the likelihood of sustained practice delivery for any of the six EBPs. Our model controlled for whether a therapist started claiming for PEI in the first year of PEI rollout or later. Starting with workforce characteristics as predictors, social workers, trainees, psychiatrists, counselors, therapists of other disciplines (e.g., case managers, psychologists), and rehabilitation professionals at the time of their first claim were all more likely to discontinue delivery than MFTs. Particularly striking are the hazard ratios for trainees and psychiatrists, who exhibit nearly twice as much risk of discontinuing delivery (94.1 and 70.8%) as MFTs. These findings suggest that allocating EBP training resources—at least for these six EBPs—toward temporary employees may represent shorter-term investments.

We found that therapists whose primary language was Spanish were at a significantly lower risk of EBP discontinuation. This group represents 34.8% of the therapist workforce represented in the data. The finding suggests that therapists who are prepared to serve the large proportion of non-English, Spanish speakers in the County system are retained in the EBP delivery workforce. The results indicate that efforts to recruit bilingual, bicultural mental health providers may provide excellent returns on EBP-training investments in individuals who are best able to reach typically underserved populations.

Not surprisingly, making more claims per day (i.e., greater volume of therapist PEI claims) was associated with a *decreased* risk of discontinuing delivery; however, serving more unique clients per month was associated with an *increased* risk of discontinuation. These somewhat contrary findings may relate to therapist burnout. Indeed, past research has shown that a high caseload is associated with an increased burnout [e.g., Ref. (29, 45)]. An increased number of unique clients controlling for the number of units of service delivered daily may translate to increased requirements for documentation and outcome monitoring with each additional unique client served. By contrast, an overall higher volume of EBP delivery may facilitate greater mastery that contributes to a longer continued use by therapists.

Therapists who billed for more EBPs or at multiple agencies were at a lower risk of discontinuing delivery of any of the six EBPs. This finding is encouraging, as it suggests that even when workforce turnover occurs at the agency level, there may be recapture of EBP-training investments at the *system* level. Relative to therapists who made the most claims to TF-CBT, therapists who made the most claims to CPP, TP, MAP, or SS were all at a lower risk of discontinuing any EBP delivery. By contrast, therapists who primarily delivered CBITS exhibited a much higher risk (more than twice) than therapists who primarily delivered TF-CBT. The latter finding is unsurprising given the lower sustainment of CBITS relative to TF-CBT documented in our prior work (15).

With respect to client case-mix characteristics, therapists with a higher proportion of Hispanic clients were at a lower risk of discontinuing any EBP delivery when compared to therapists with a higher proportion of clients of other ethnicities. These findings suggest that when a given therapists’ caseload primarily resembles the most prevalent client profiles served in a given system (46) (i.e., younger, Hispanic/Latino children presenting with mood/anxiety disorders served in school settings), EBP delivery retention is more likely. Having older child clients on average was also associated with a higher risk for therapists to discontinue practice delivery. This may be explained by the fact that the coverage of the EBPs under study predominantly targets children rather than adolescents. We did not find an association between the risk of discontinuation and caseload representation of youth with admission diagnoses other than mood, anxiety, conduct, and trauma problems targeted by the six EBPs. However, the average representation of these problems was low on average in the sample.

With respect to organizational characteristics, the centralization of the agency and the primary service setting type were associated with a risk of delivery discontinuation. First, therapists at agencies where services were primarily school-based had longer tenures of sustaining EBP delivery compared to therapists at office-based sites. This finding may suggest that therapists primarily delivering care in settings with fewer access barriers may show more longevity in EBP implementation. Second, claiming at a multisite agency was associated with a higher risk of discontinuing EBP delivery than being at a single-site agency. Centralized, single-site agencies tend to also be smaller than multisite agencies. Previous findings from the same system context have suggested that larger agencies installed more systematic strategies at multiple levels (i.e., organization, therapist, client) to support initial EBP implementation (38). However, the current findings may indicate that the greater resources put in place by larger agencies may not ensure EBP sustainment at the therapist level. However, this is in contrast to research demonstrating turnover to be lower where employees are more embedded in their job and work in larger organizations (47).

Some limitations of the present study must be noted. The limited time frame of current data precluded analysis of sustainment beyond 19 fiscal quarters; indeed, 35.5% of therapists continued to bill for one of the six EBPs within the final fiscal quarter of our analysis. However, survival analysis accounts for those therapists who are considered to be “censored” to produce a reliable model. In addition, these data do not shed light on whether therapists discontinued delivering PEI EBPs *altogether* or whether they might have initiated or continued to deliver PEI EBPs other than the six examined in this study. Relatedly, this study was only able to examine the sustainment of these six approved PEI practices, as they were the only ones initially selected by the LACDMH for implementation support (38). Furthermore, we were unable to track therapist migration to mental health agencies not billing to PEI; it is plausible that these therapists continued to deliver one of the six EBPs at another agency (e.g., a private practice, an agency outside of Los Angeles County) not represented in the LACDMH claims data. In addition, we were not able to control for other variables that have been associated with sustainment, such as community readiness, the extent to which program staff received support and assistance, and other contextual factors [e.g., Ref. (20, 48)]. While we are unable to track specific instances of “turnover” *per se*, our finding that therapists who provided services at more than one agency had better survival odds indicated that turnover across agencies may not be inconsistent with therapist-level sustainment of EBP delivery in this context. A limitation inherent to using administrative claims data is that we infer “delivery” when the data itself only truly indicate “billing.” Claims data also do not indicate whether a practice was delivered with fidelity. In addition, importantly, the claims data included in the current study do not represent the entirety of a therapist’s practice, that is, these data only represent that therapist’s administrative claims for these six EBPs for children or transition-age youth. Therapists likely served other many other children through different funding sources and/or other EBPs covered under the PEI program, whereas some therapists may also have served clients of other age ranges.

Despite these limitations, this study has important implications for system-driven implementation efforts. This study illustrates the novel contribution of applying survival analysis methods to administrative claims data to examine returns on system-level investments in workforce training. The findings provide a benchmark for continued therapist EBP delivery within a system (vs. individual organizations). Furthermore, the findings suggest potential mutable factors to target in sustainment interventions. For example, the findings highlight that strategic assignment of therapists to EBP training should be based on maximizing fit between the EBP and the therapist’s existing case mix. Likewise, the findings also underscore the importance of relevance-mapping approaches to the system- and agency-level selection of EBPs for adoption with the goal of long-term sustainment (14).

## Author Contributions

LB-F and AL contributed to the study design/framework, outlined the introduction, and drafted the discussion. CZ contributed to the study design, research methodology, performed the analyses, and drafted the introduction, methods, results, and discussion. NS contributed to the study aims and methods. DS, SR, and GA contributed to the research methodology and manuscript editing. DI-G and LB facilitated access to the data used in this study and contributed to the interpretation of findings. All authors have reviewed this manuscript.

## Conflict of Interest Statement

The authors declare that the research was conducted in the absence of any commercial or financial relationships that could be construed as a potential conflict of interest. The reviewer KH and handling Editor declared their shared affiliation.
